# Blood-Based Tau as a Biomarker for Early Detection and Monitoring of Alzheimer’s Disease: A Systematic Review and Meta-Analysis

**DOI:** 10.3390/ijms262110330

**Published:** 2025-10-23

**Authors:** Ka Young Kim, Ki Young Shin, Keun-A Chang

**Affiliations:** 1 Department of Nursing, College of Nursing, Gachon University, Incheon 21936, Republic of Korea; kykim@gachon.ac.kr; 2 Neuroscience Research Institute, Gachon University, Incheon 21565, Republic of Korea; 3 Bio-MAX Institute, Seoul National University, Seoul 08826, Republic of Korea; 4 Department of Pharmacology, College of Medicine, Gachon University, Incheon 21999, Republic of Korea

**Keywords:** Alzheimer’s disease, tau protein, blood-based biomarkers, mild cognitive impairment, tau PET, early diagnosis

## Abstract

Alzheimer’s disease (AD), the most prevalent form of dementia, is a progressive neurodegenerative disorder characterized by cognitive decline and memory loss, ultimately leading to loss of independence and reduced quality of life. Since current treatments are most effective in early stages, the development of reliable and noninvasive biomarkers for early diagnosis and monitoring is crucial. Abnormal tau protein aggregation is a key pathological hallmark of AD, disrupting neuronal integrity, accelerating progression, and associating closely with cognitive decline and the transition to mild cognitive impairment, a prodromal stage of AD. Currently, tau pathology is evaluated mainly by cerebrospinal fluid analysis and tau positron emission tomography (tau PET), which are invasive or costly, limiting their clinical applicability. This systematic review and meta-analysis synthesized evidence on tau as a blood-based biomarker for dementia, with emphasis on its relationship to tau PET, the gold standard for in vivo tau assessment. Findings indicate that elevated plasma tau levels such as p-tau181, p-tau217 and p-tau231 consistently reflect brain tau pathology, supporting their role as surrogate markers. Large-scale longitudinal validation is warranted to establish blood-based tau as a practical, accessible tool for early detection and disease monitoring, thereby improving therapeutic outcomes in AD.

## 1. Introduction

Alzheimer’s disease (AD), the most prevalent form of dementia, is a progressive neurodegenerative disorder characterized by gradual cognitive decline and memory loss, ultimately resulting in loss of independence and reduced quality of life [1,2]. With its rapidly increasing global prevalence, AD imposes a substantial medical, social, and economic burden, underscoring the urgent need for strategies that enable earlier diagnosis and timely intervention [3].

Among the pathological hallmarks of AD, the abnormal aggregation and deposition of tau protein are particularly critical [4]. Aberrant tau accumulation disrupts neuronal integrity, accelerates disease progression, and is strongly associated with cognitive decline and development of mild cognitive impairment (MCI), a prodromal stage of AD [5,6]. Currently, tau pathology is primarily assessed using cerebrospinal fluid (CSF) analysis and tau positron emission tomography (tau PET) [7]. Although these modalities provide invaluable insights for clinical and research applications, their invasiveness, high cost, and limited accessibility restrict widespread use [8,9].

Therefore, blood-based biomarkers have emerged as promising alternatives for detecting and monitoring [10]. They are safe, cost-effective, and easily accessible, allowing for repeated measurements, longitudinal tracking, and large-scale applications [11]. Since tau abnormalities occur years before the onset of overt dementia [12], assessing blood tau levels in individuals with MCI may enable earlier diagnosis, risk stratification, and intervention [13]. If validated, blood tau could complement or reduce the reliance on CSF and tau-PET-based imaging, thereby enhancing diagnostic accuracy, improving accessibility, and supporting more effective disease monitoring and prognosis in AD [14,15].

The present systematic review and meta-analysis critically evaluated the current evidence on tau proteins (p-tau181, p-tau217 and p-tau231) as a blood-based biomarker for the early detection of dementia, with particular focus on its concordance with tau PET, which is the current gold standard for in vivo tau assessment.

## 2. Materials and Methods

### 2.1. Literature Search

A literature search was conducted using PubMed, Embase, Web of Science, and the Cochrane Library. The search covered articles published up to 20 February 2025, and was independently performed by two researchers (K.Y. Kim & K.-A. Chang). The search strategy used combinations of the following keywords: (Alzheimer) AND (“mild cognitive impairment” OR MCI) AND tau AND (pet OR “positron emission tomography” OR “positron emission tomography” OR “scintigraphy” OR “tracer”) AND (blood OR plasma OR serum). Ethical approval and obtaining patient consent were not required as this study is a systematic review and meta-analysis of previously published studies. The literature search and study selection process followed the Preferred Reporting Items for Systematic Reviews and Meta-Analyses 2020 guidelines.

### 2.2. Eligibility Criteria

The inclusion criteria were as follows: (a) studies in which the diagnosis of AD or MCI was based on the grouping criteria defined in each original study; (b) studies that included cognitively unimpaired individuals as controls and patients with MCI and AD; (c) studies reporting plasma tau biomarkers (total tau, p-tau181, p-tau217, or p-tau231) and tau PET imaging outcomes; and (d) studies published in English. The exclusion criteria were as follows: (a) reviews, case reports, conference abstracts, or animal studies; (b) studies without available quantitative data on tau biomarkers; and (c) duplicate or overlapping datasets.

### 2.3. Data Extraction and Quality Assessment

Two authors independently extracted data from each included study, and discrepancies were resolved by discussion or adjudication by a third author. The extracted information included the first author, year of publication, country, sample size, sex distribution, mean age, cognitive assessment tools (e.g., MMSE, CDR, MoCA), plasma tau biomarkers (total tau, p-tau181, p-tau217, p-tau231), and tau PET outcomes (tracer type, SUVR values, or correlation results). Study quality was assessed using the risk-of-bias tool in Review Manager 5.4, which evaluates methodological rigor and applicability. The risk of bias was categorized as “low,” “high,” or “uncertain” according to predefined criteria.

**Figure 1:**
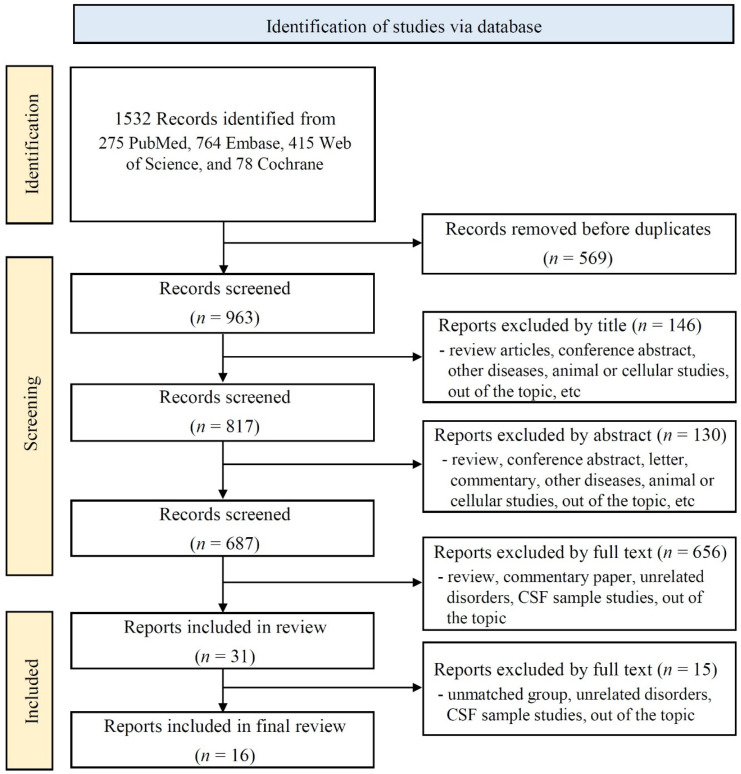
Flow chart.

A total of 1532 records were initially retrieved from the database searches, including 78 from the Cochrane Library, 764 from Embase, 275 from PubMed, and 415 from Web of Science (Figure 1). After removing the duplicates, 963 records were retained for screening. Of these, 817 were identified after title screening, 687 after abstract screening, and 31 after full-text review. Following a thorough review of the full texts, 16 studies met the eligibility criteria and were included in the quantitative synthesis.

### 2.4. Effect Size and Statistical Analysis

Meta-analyses were performed using the Review Manager 5.4 software. Effect size estimates were calculated as SMDs with corresponding 95% CIs. SMD was chosen to enable the appropriate synthesis of biomarker data reported across studies using different measurement scales and assay methods, thereby allowing for the direct comparability of results. Positive SMD values indicate higher biomarker levels in the disease group than in the reference group. Pooled analyses were conducted separately for each plasma tau biomarker (p-tau181, p-tau217, and p-tau231), as well as an overall pooled analysis across all plasma tau markers. For tau PET, pooled analyses were conducted according to tracer type, including ^18^F-flortaucipir and ^18^F-MK6240, and SUVRs were synthesized. Analyses were restricted to data that were suitable for quantitative synthesis. Heterogeneity across studies was assessed using Cochran’s Q (χ^2^) test and quantified with the I^2^ statistic. A fixed-effects model was applied when heterogeneity was negligible (I^2^ < 50%, *p* > 0.1), whereas a random-effects model was applied when substantial heterogeneity was observed (I^2^ ≥ 50%, *p* ≤ 0.1). In addition, because heterogeneity statistics are less reliable when only a small number of studies are included, a random-effects model was conservatively applied to the subgroups of three or fewer studies. Subgroup and sensitivity analyses were performed where appropriate (Supplementary Figure S1), and publication bias was evaluated using funnel plots (Supplementary Figure S2).

## 3. Results

### 3.1. Study Characteristics

Table 1 summarizes the main characteristics of the 16 included studies. This research was conducted in several countries, including Australia, Canada, China, Sweden, Switzerland, and the United States. In line with the inclusion criteria, the studies included controls, MCI, and AD groups. Group classifications were based on cognitive assessment tools such as the Mini-Mental State Examination (MMSE), Clinical Dementia Rating (CDR), and Montreal Cognitive Assessment (MoCA), with the corresponding cognitive scores presented in Table 1. Additional variables included sample size, sex distribution, proportion of female participants, and mean age.

### 3.2. Tau Biomarker Findings

Table 2 summarizes the plasma tau biomarkers, including total tau and phosphorylated isoforms (p-tau181, p-tau217, and p-tau231), across the control, MCI, and AD groups. Each study reported the statistical significance of the group comparisons (MCI vs. control, AD vs. control, and AD vs. MCI). Tau PET outcomes were also obtained using specified tracers. Studies have reported either the standardized uptake value ratio (SUVR) or the correlation and statistical significance of tau deposition. Tau-PET imaging employs both first-generation tracers, such as ^18^F-flortaucipir, and second-generation tracers, such as ^18^F-MK6240 and ^18^F-RO948.

### 3.3. Quality Assessment

**Figure 2:**
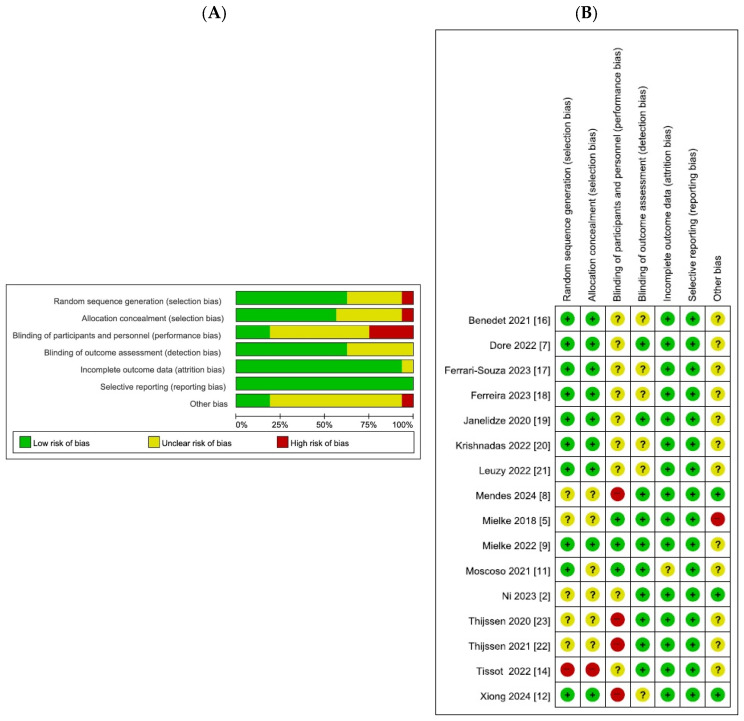
Quality assessment. Bar chart and bar chart of bias risk assessment. Different colors (green, red, yellow) and symbols (“+”, “−“,” “?”) represent “low risk bias,” “high risk bias,” and “unclear,” respectively. The quality assessment chart includes the percentage of bias risk at each level graph, and the level of applicability concerns for each specific item in the study. (**A**) Risk of bias graph (**B**) Risk of bias summary.

Figure 2 presents the quality assessment of the included non-randomized studies, evaluated using the Risk-of-Bias Assessment Tool for Non-randomized Studies. Most studies showed a low risk of bias in participant selection and exposure measurements, reflecting clearly defined eligibility criteria and standardized biomarker assessment methods. The outcome assessment was also generally rated as low risk, as biomarker assays and PET imaging analyses were conducted using validated protocols. Incomplete outcome data and selective reporting were mostly judged as low-risk, although a few studies were classified as unclear owing to missing subgroup data or a lack of prespecified reporting plans. By contrast, the domain of confounding variables demonstrated a higher proportion of unclear or high-risk ratings, as adjustment for key covariates such as age, sex, or APOE ε4 status was often limited. Overall, the methodological quality of the included studies was acceptable, with a risk of bias generally low to moderate across most domains.

### 3.4. Meta-Analysis of Plasma Tau Biomarkers

**Figure 3:**
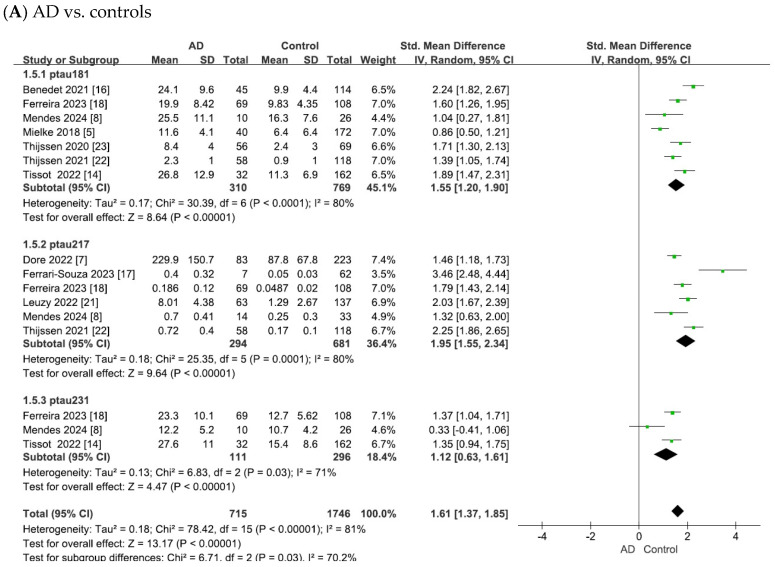

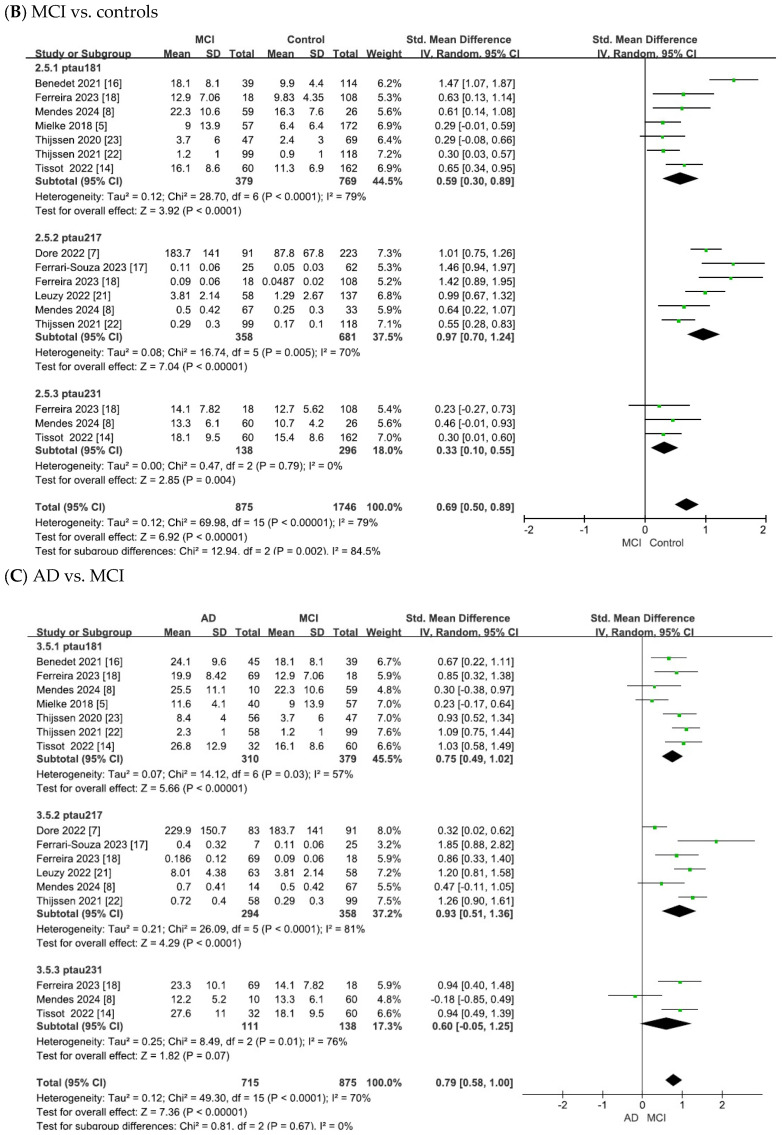
Forest plots of meta-analysis of plasma tau biomarkers (p-tau181, p-tau217, and p-tau231).

The results of this meta-analysis are summarized in Figure 3. Pooled analyses were performed separately for p-tau181, p-tau217, and p-tau231. Group comparisons were conducted between the (A) AD and control, (B) MCI and control, and (C) AD and MCI groups.

In the comparison of AD vs. controls (Figure 3), substantial heterogeneity was observed across studies (p-tau181: χ^2^ = 30.39, *p* < 0.0001, I^2^ = 80%; p-tau217: χ^2^ = 25.35, *p* < 0.0001, I^2^ = 80%; p-tau231: χ^2^ = 6.83, *p* = 0.03, I^2^ = 71%). Using random-effects models, pooled standardized mean differences (SMDs) showed significant elevations in AD: p-tau181 (SMD = 1.55, 95% confidence intervals (CI) = [1.20, 1.90], Z = 8.64, *p* < 0.00001), p-tau217 (SMD = 1.95, 95% CI = [1.55, 2.34], Z = 9.64, *p* < 0.00001), and p-tau231 (SMD = 1.12, 95% CI = [0.63, 1.61], Z = 4.47, *p* < 0.00001). In all cases, the differences were statistically significant, with elevated plasma tau levels in patients with AD compared with controls.

As shown in Figure 3B, the meta-analysis focused on the comparisons between patients with MCI and controls. The heterogeneity test of the included studies indicated substantial heterogeneity for plasma tau biomarkers (p-tau181: χ^2^ = 28.70, *p* < 0.0001, I^2^ = 79%; p-tau217: χ^2^ = 16.74, *p* = 0.005, I^2^ = 70%; p-tau231: χ^2^ = 0.47, *p* = 0.79, I^2^ = 0). Although the heterogeneity was low for p-tau231 (I^2^ = 0%), the number of available studies was small (n = 3). Therefore, in line with the I^2^ thresholds and by adopting a conservative approach for comparisons with limited study numbers, random-effects models were applied across all analyses. This yielded combined SMDs of 0.59 (95% CI = [0.30, 0.89], Z = 3.92, *p* < 0.0001) for p-tau181, 0.97 (95% CI = [0.70, 1.24], Z = 7.04, *p* < 0.00001) for p-tau217, and 0.33 (95% CI = [0.10, 0.55], Z = 2.85, *p* = 0.004) for p-tau231. In all cases, the differences were statistically significant, with elevated plasma tau levels in patients with MCI compared to controls.

As shown in Figure 3C, the meta-analysis focused on comparisons between patients with AD and those with MCI. The heterogeneity test of the included studies indicated substantial heterogeneity for plasma tau biomarkers (p-tau181: χ^2^ = 14.12, *p* = 0.03, I^2^ = 57%; p-tau217: χ^2^ = 26.09, *p* < 0.0001, I^2^ = 81%; p-tau231: χ^2^ = 8.49, *p* = 0.01, I^2^ = 76%). Random-effects models were therefore applied to pool the effect sizes, yielding combined SMDs of 0.75 (95% CI = [0.49, 1.02], Z = 5.66, *p* < 0.00001) for p-tau181, 0.93 (95% CI = [0.51, 1.36], Z = 4.29, *p* < 0.0001) for p-tau217, and 0.60 (95% CI = [−0.05, 1.25], Z = 1.82, *p* = 0.07) for p-tau231. In all cases except for p-tau231, the differences were statistically significant, with plasma tau levels being elevated in AD compared to MCI.

Furthermore, the overall pooled results across all plasma tau biomarkers for the AD vs. control, MCI vs. control, and AD vs. MCI group comparisons are presented in Supplementary Figure S3.

### 3.5. Meta-Analysis of Tau PET Biomarkers

**Figure 4:**
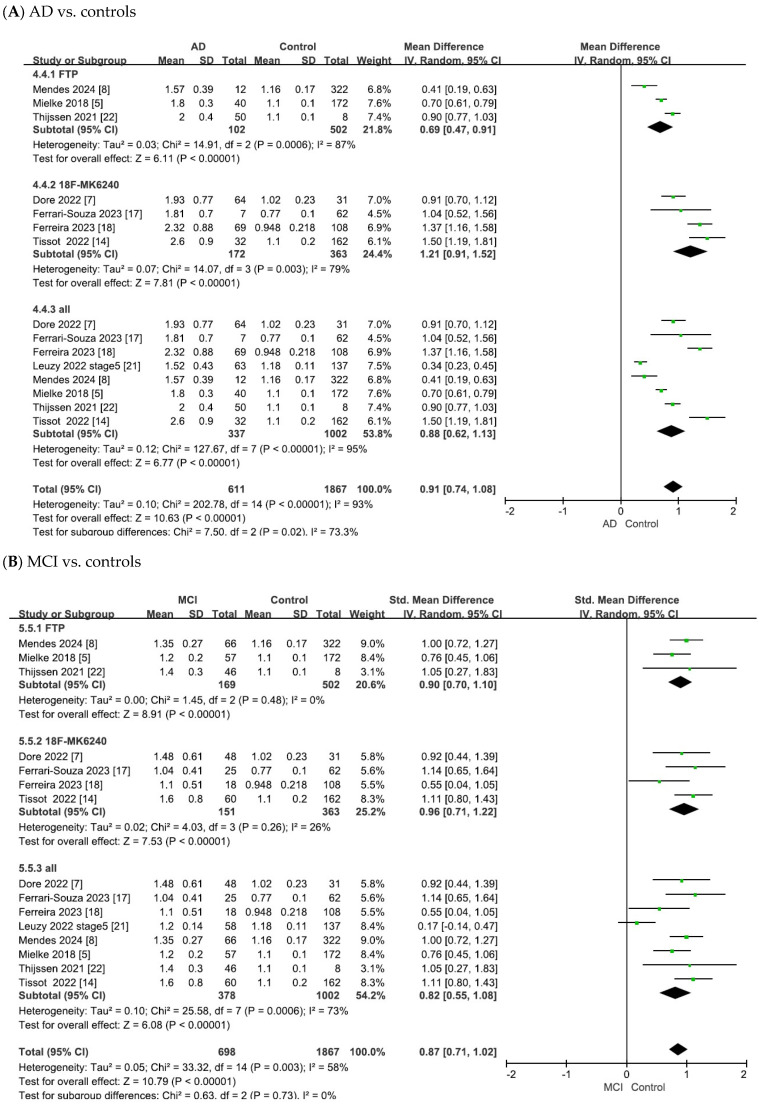

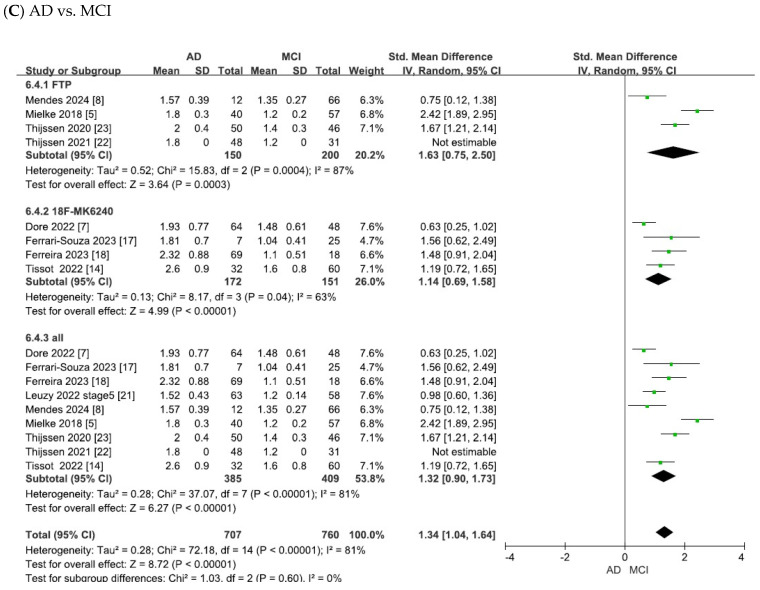
Forest plots of meta-analysis of tau PET biomarkers (^18^F-flortaucipir (FTP), ^18^F-MK6240, and overall).

The meta-analysis results for tau PET biomarkers are presented in Figure 4. For tau PET, pooled analyses were performed separately for ^18^F-flortaucipir and ^18^F-MK6240 as well as an overall pooled analysis that included ^18^F-flortaucipir, ^18^F-MK6240, and ^18^F-RO948. Figure 4 summarizes the pooled effect sizes for tau PET biomarkers across studies, presenting group comparisons between (A) AD and controls, (B) MCI and controls, and (C) AD and MCI groups.

As shown in Figure 4A, the heterogeneity test of the included studies indicated substantial heterogeneity for tau PET biomarkers (^18^F-flortaucipir: χ^2^ = 14.91, *p* = 0.0006, I^2^ = 87%; ^18^F-MK6240: χ^2^ = 14.07, *p* = 0.003, I^2^ = 79%; overall: χ^2^ = 127.67, *p* < 0.00001, I^2^ = 95%). Random-effects models were therefore applied to pool the effect sizes, yielding combined SMDs of 0.69 (95% CI = [0.47, 0.91], Z = 6.11, *p* < 0.00001) for ^18^F-flortaucipir, 1.21 (95% CI = [0.91, 1.52], Z = 7.81, *p* < 0.00001) for ^18^F-MK6240 and 0.88 (95% CI = [0.62, 1.13], Z = 6.77, *p* < 0.00001) for the overall pooled analysis. In all cases, the differences were statistically significant, with tau PET SUVR values being elevated in patients with AD compared with controls.

As shown in Figure 4B, the meta-analysis focused on the comparisons between patients with MCI and controls. The heterogeneity test of the included studies indicated substantial heterogeneity for tau PET biomarkers (^18^F-flortaucipir: χ^2^ = 1.45, *p* = 0.48, I^2^ = 0; ^18^F-MK6240: χ^2^ = 4.03, *p* = 0.26, I^2^ = 26%; overall: χ^2^ = 25.58, *p* = 0.0006, I^2^ = 73%). To adopt a conservative approach in synthesizing evidence from the small number of observational studies, random-effects models were applied across all analyses. This yielded combined SMDs of 0.90 (95% CI = [0.70, 1.10], Z = 8.91, *p* < 0.00001) for ^18^F-flortaucipir, 0.96 (95% CI = [0.71, 1.22], Z = 7.53, *p* < 0.00001) for ^18^F-MK6240 and 0.82 (95% CI = [0.55, 1.08], Z = 6.08, *p* < 0.00001) for the overall pooled analysis. In all cases, the differences were statistically significant, with tau PET SUVR values being elevated in patients with MCI compared to controls.

As shown in Figure 4C, the meta-analysis focused on comparisons between patients with AD and those with MCI. The heterogeneity test of the included studies indicated substantial heterogeneity for tau PET biomarkers (^18^F-flortaucipir: χ^2^ = 15.83, *p* = 0.0004, I^2^ = 87%; ^18^F-MK6240: χ^2^ = 8.17, *p* = 0.04, I^2^ = 63%; overall: χ^2^ = 37.07, *p* < 0.00001, I^2^ = 81%). Random-effects models were therefore applied to pool the effect sizes, yielding combined SMDs of 1.63 (95% CI = [0.75, 2.50], Z = 3.64, *p* = 0.00003) for ^18^F-flortaucipir, 1.14 (95% CI = [0.69, 1.58], Z = 4.99, *p* < 0.00001) for ^18^F-MK6240 and 1.32 (95% CI = [0.90, 1.73], Z = 6.27, *p* < 0.00001) for the overall pooled analysis. In all cases, the differences were statistically significant, with tau PET SUVR values being elevated in AD compared to MCI.

### 3.6. Meta-Analysis Matching Plasma Tau Isoforms with Tau PET

**Figure 5:**
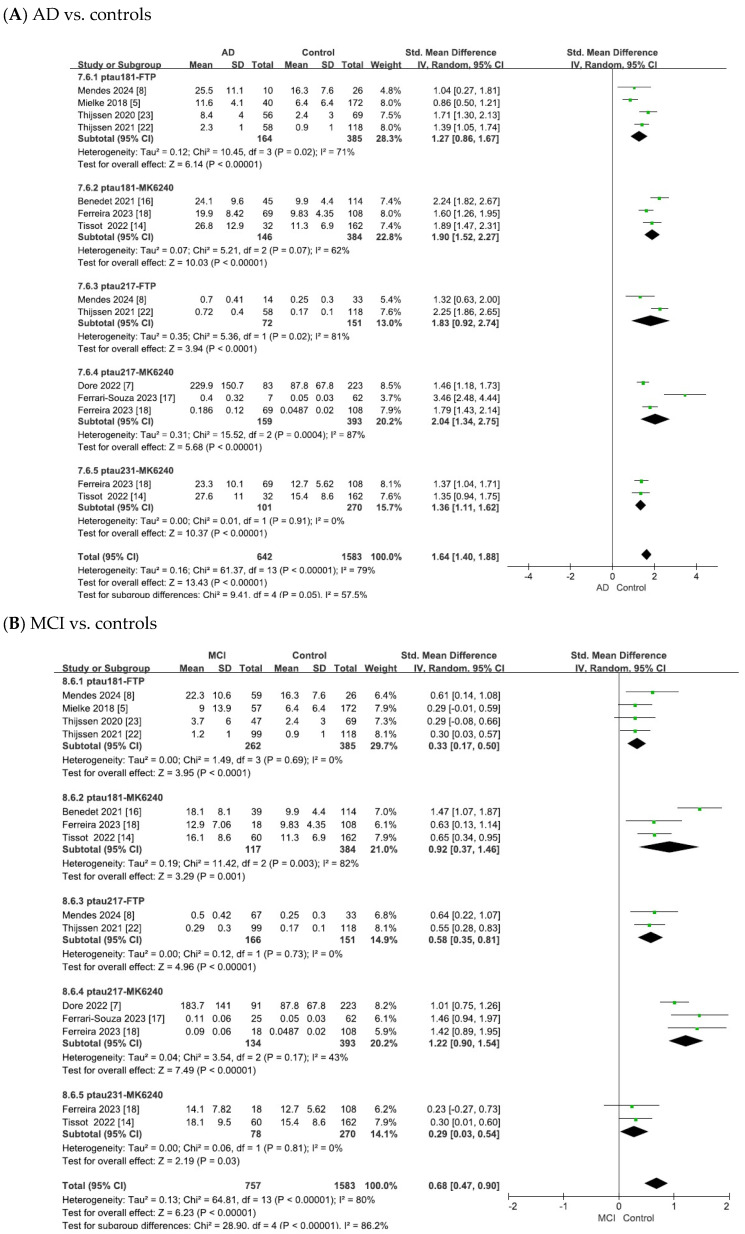

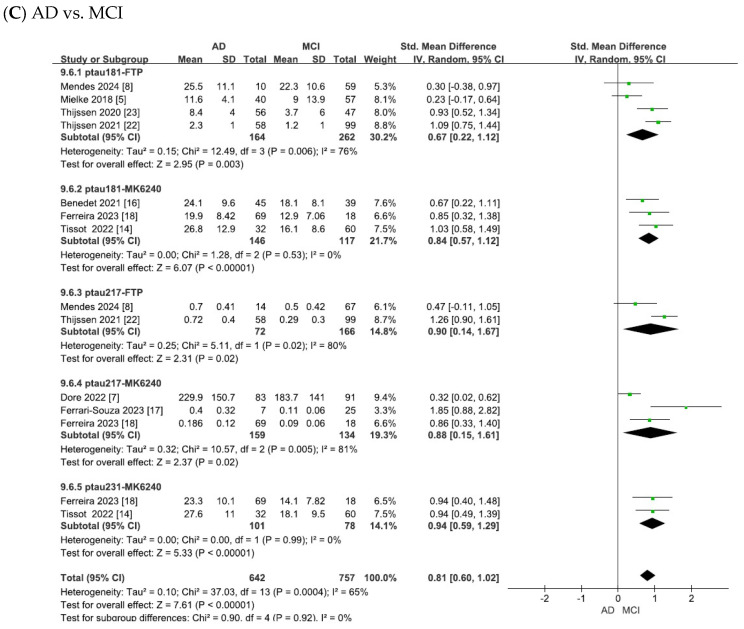
Forest plots of meta-analysis matching plasma tau isoforms with tau PET tracers. Abbreviations: FTP, ^18^F-flortaucipir.

The meta-analysis results for the matched plasma tau isoforms and tau PET tracers are shown in Figure 5. Paired comparisons were performed for p-tau181 with ^18^F-flortaucipir and ^18^F-MK6240, for p-tau217 with ^18^F-flortaucipir and ^18^F-MK6240, and for p-tau231 with ^18^F-MK6240. Figure 5 summarizes the pooled effect sizes for these plasma–PET combinations across the studies, presenting group comparisons between (A) AD and controls, (B) MCI and controls, and (C) AD and MCI groups.

As shown in Figure 5A, the heterogeneity test of the included studies indicated substantial heterogeneity for the matched plasma–PET biomarker pairs (p-tau181 with ^18^F-flortaucipir: χ^2^ = 10.45, *p* = 0.02, I^2^ = 71%; p-tau181 with ^18^F-MK6240: χ^2^ = 5.21, *p* = 0.07, I^2^ = 62%; p-tau217 with ^18^F-flortaucipir: χ^2^ = 5.36, *p* = 0.02, I^2^ = 81%; p-tau217 with ^18^F-MK6240: χ^2^ = 15.52, *p* = 0.0004, I^2^ = 87%; p-tau231 with ^18^F-MK6240: χ^2^ = 0.01, *p* = 0.91, I^2^ = 0). To adopt a conservative approach in synthesizing evidence from the small number of observational studies, random-effects models were applied across all analyses. This yielded combined SMDs of 1.27 (95% CI = [0.86, 1.67], Z = 6.14, *p* < 0.00001) for p-tau181 with ^18^F-flortaucipir, 1.90 (95% CI = [1.52, 2.27], Z = 10.03, *p* < 0.00001) for p-tau181 with ^18^F-MK6240, 1.83 (95% CI = [0.92, 2.74], Z = 3.94, *p* < 0.00001) for p-tau217 with ^18^F-flortaucipir, 2.04 (95% CI = [1.34, 2.75], Z = 5.68, *p* < 0.00001) for p-tau217 with ^18^F-MK6240, and 1.36 (95% CI = [1.11, 1.62], Z = 10.37, *p* < 0.00001) for p-tau231 with ^18^F-MK6240. In all cases, the differences were statistically significant, with tau PET SUVR values showing significant associations with the corresponding plasma tau isoforms in patients with AD compared to controls.

As shown in Figure 5B, the meta-analysis focused on the comparisons between patients with MCI and controls. The heterogeneity test of the included studies indicated substantial heterogeneity for the matched plasma–PET biomarker pairs (p-tau181 with ^18^F-flortaucipir: χ^2^ = 1.49, *p* = 0.69, I^2^ = 0; p-tau181 with ^18^F-MK6240: χ^2^ = 11.42, *p* = 0.003, I^2^ = 82%; p-tau217 with ^18^F-flortaucipir: χ^2^ = 0.12, *p* = 0.73, I^2^ = 0; p-tau217 with ^18^F-MK6240: χ^2^ = 3.54, *p* = 0.17, I^2^ = 43%; p-tau231 with ^18^F-MK6240: χ^2^ = 0.06, *p* = 0.81, I^2^ = 0). To adopt a conservative approach in synthesizing evidence from the small number of observational studies, random-effects models were applied across all analyses. This yielded combined SMDs of 0.33 (95% CI = [0.17, 0.50], Z = 3.95, *p* < 0.0001) for p-tau181 with ^18^F-flortaucipir, 0.92 (95% CI = [0.37, 1.46], Z = 3.29, *p* = 0.001) for p-tau181 with ^18^F-MK6240, 0.58 (95% CI = [0.35, 0.81], Z = 4.96, *p* < 0.00001) for p-tau217 with ^18^F-flortaucipir, 1.22 (95% CI = [0.90, 1.54], Z = 7.49, *p* < 0.00001) for p-tau217 with ^18^F-MK6240, and 0.29 (95% CI = [0.03, 0.54], Z = 2.19, *p* = 0.03) for p-tau231 with ^18^F-MK6240. In all cases, the differences were statistically significant, with tau PET SUVR values showing significant associations with the corresponding plasma tau isoforms in patients with MCI compared to controls.

As shown in Figure 5C, the meta-analysis focused on comparisons between patients with AD and those with MCI. The heterogeneity test of the included studies indicated substantial heterogeneity for the matched plasma–PET biomarker pairs (p-tau181 with ^18^F-flortaucipir: χ^2^ = 12.49, *p* = 0.006, I^2^ = 76%; p-tau181 with ^18^F-MK6240: χ^2^ = 1.28, *p* = 0.53, I^2^ = 0; p-tau217 with ^18^F-flortaucipir: χ^2^ = 5.11, *p* = 0.02, I^2^ = 80%; p-tau217 with ^18^F-MK6240: χ^2^ = 10.57, *p* = 0.005, I^2^ = 81%; p-tau231 with ^18^F-MK6240: χ^2^ = 0.00, *p* = 0.99, I^2^ = 0). To adopt a conservative approach in synthesizing evidence from the small number of observational studies, random-effects models were applied across all analyses. This yielded combined SMDs of 0.67 (95% CI = [0.22, 1.12], Z = 2.95, *p* = 0.003) for p-tau181 with ^18^F-flortaucipir, 0.84 (95% CI = [0.57, 1.12], Z = 6.07, *p* < 0.00001) for p-tau181 with ^18^F-MK6240, 0.90 (95% CI = [0.14, 1.67], Z = 2.31, *p* = 0.02) for p-tau217 with ^18^F-flortaucipir, 0.88 (95% CI = [0.15, 1.61], Z = 2.37, *p* = 0.02) for p-tau217 with ^18^F-MK6240, and 0.94 (95% CI = [0.59, 1.29], Z = 5.33, *p* < 0.00001) for p-tau231 with ^18^F-MK6240. In all cases, the differences were statistically significant, with tau PET SUVR values showing significant associations with the corresponding plasma tau isoforms in AD compared to MCI.

## 4. Discussion

In this study, we demonstrated that both first- (^18^F-flortaucipir) and second-generation tau-PET tracers (^18^F-MK6240 and ^18^F-RO948), previously reported [24], can distinguish patients with AD from cognitively normal individuals and patients with MCI from both normal controls and AD patients [24]. These results support the diagnostic utility of tau PET across disease stages. Previous studies have similarly shown that tau PET reliably maps the spatial and temporal progression of tau deposition and is associated with clinical severity and neurodegeneration [25,26,27]. Therefore, our findings are consistent with the use of tau PET as a reference standard for the in vivo assessment of tau pathology.

Importantly, we also found that plasma phosphorylated tau (p-tau) isoforms (p-tau181, p-tau217, and p-tau231) were significantly elevated in patients with AD and MCI compared with controls (Figure 3), and that these changes broadly paralleled the tau-PET findings (Figure 5). This concordance supports the role of blood tau levels as peripheral biomarkers for central tau pathology. While other plasma markers such as tau, p-tau202, and p-tau181/Aβ_1–42_ ratio have also been reported to correlate with tau PET and clinical outcomes [28,29], accumulating evidence consistently validates p-tau181, p-tau217, and p-tau231 as the most promising blood-based diagnostic markers [14,30,31,32,33]. The diagnostic application of these tau isoforms in AD offers a promising alternative for overcoming the challenge of differential diagnosis in conditions such as frontotemporal dementia (FTD) and traumatic brain injury (TBI), where elevated tau levels alone are insufficient for disease discrimination. Although tau protein elevation can occur in FTD and TBI [34,35], p-tau217 appears to show relatively higher specificity for AD pathology and may outperform other isoforms in differentiating disease states [36,37]. Moreover, growing evidence indicates that longitudinal trajectories of plasma p-tau, rather than single-time-point concentrations, are more closely associated with cognitive decline and the progression of tau pathology [37]. Taken together, these results support the integration of blood tau assays as surrogate measures for imaging biomarkers, bridging the gap between research tools and routine clinical practice.

These findings have important clinical implications. First, blood tau offers a minimally invasive and affordable alternative to CSF assays and tau PET, which, despite its diagnostic value, remains costly, invasive, and less accessible in routine care [8,23]. Blood sampling is safe, repeatable, and feasible for longitudinal monitoring, making it suitable for large-scale population screening [38,39]. Thus, blood tau could help expand biomarker-based diagnoses, especially in primary care and community health settings where advanced imaging modalities are not widely available.

Second, the detection of elevated blood tau levels in MCI highlights its potential for diagnosis [40,41]. Tau pathology arises years before dementia onset [42,43] and identifying tau abnormalities during this prodromal stage could allow for earlier risk stratification, trial enrollment, and therapeutic interventions [44]. Detecting the disease at this stage is critical, as interventions may be more effective before irreversible neurodegeneration occurs.

Third, among the most actively studied phosphorylated tau species—p-tau181, p-tau217, and p-tau231—each appears to offer complementary strengths and may have distinct roles in AD assessment. In particular, p-tau217 has shown comparatively strong performance in several multicenter, head-to-head evaluations, suggesting it could be suitable for pathology triage and clinical trial enrichment [45]. By contrast, p-tau231 (along with p-tau217) may be especially sensitive to very early Aβ-related changes, potentially aiding identification at preclinical stages [46]. p-tau181, supported by broader availability across platforms and a substantial evidence base, remains a practical option for wider screening and longitudinal follow-up [47]. Several studies have proposed threshold values that may help differentiate AD from other neurodegenerative conditions and cognitively unimpaired individuals. For instance, plasma p-tau181 levels exceeding approximately 2.2 pg/mL have been associated with increased probability of amyloid-β and tau pathology [9]. Plasma p-tau217 has shown relatively high diagnostic performance, with an optimal cut-off around 0.15 pg/mL identified in ROC analyses distinguishing AD from non-AD dementias [48]. In addition, plasma p-tau231, measured using an in-house Simoa assay, has been reported to exhibit a threshold near 17.65 pg/mL, showing potential to reflect early amyloid-related changes and to distinguish AD from healthy controls [14,49]. These findings collectively indicate that while assay methodology and cohort characteristics may influence the specific thresholds observed, plasma p-tau species represent valuable biomarkers warranting further comparative and longitudinal evaluation.

In addition to its diagnostic applications, blood tau may be valuable for monitoring disease progression and treatment response. However, it is unlikely that plasma tau will completely replace tau-PET, which remains the gold standard for in vivo assessment of tau pathology. Tau-PET provides valuable spatial and quantitative information on tau deposition throughout the brain, allowing direct visualization of disease stage and regional involvement [27,50]. In contrast, blood-based tau biomarkers, including p-tau181, p-tau217, and p-tau231, offer complementary but distinct advantages in terms of accessibility, scalability, and longitudinal monitoring [46,51]. Longitudinal studies have indicated that plasma tau levels track cognitive decline and conversion to dementia [52,53]. Therefore, blood-based biomarkers could help disease monitoring, guide clinical decisions, and improve clinical trial designs by refining patient stratification and identifying those most likely to benefit from emerging therapies.

Despite these strengths, this study has a few limitations. Considering the high heterogeneity, possible cohort overlap, variability in analytic approaches, and limited longitudinal evidence, blood tau remains a preliminary biomarker; standardized methods, prospective validation and longitudinal studies are needed. First, the analysis was restricted to data from the included studies, which limits the generalizability of the results. Second, overlapping authorship and cohort data may have introduced a bias. Third, although blood tau showed generally concordance with tau PET, validation in large, ethnically diverse, and longitudinal cohorts is required to confirm its diagnostic accuracy and predictive value. Fourth, although we performed leave-one-out sensitivity analyses, substantial heterogeneity remained in several pooled analyses, which may reflect differences in study populations, tracer types, and assay methods across studies. Fifth, it can also be used in combination with tau proteins and other biomarkers. For examples, *microtubule-associated protein tau* (*MAPT*) mRNA warrants attention. Although direct evidence demonstrating an increase in circulating *MAPT* mRNA levels in Alzheimer’s disease is limited [54], alterations in *MAPT* transcript structure have been identified in brain tissue [55]. Therefore, if further studies on blood-derived *MAPT* mRNA are accumulated, combined analysis of *MAPT* mRNA and blood tau protein could potentially enhance the diagnostic accuracy for AD. Finally, although blood tau is highly promising, it is unlikely to replace CSF or imaging-based approaches. Instead, multimodal biomarker strategies that combine blood, CSF, imaging, and genetic data may provide greater diagnostic and prognostic accuracy. Future studies should validate blood tau in large, longitudinal, and ethnically diverse cohorts to confirm its diagnostic accuracy and predictive value. Standardized data reporting and integration with CSF, imaging, and genetic biomarkers will help reduce bias and improve diagnostic precision, while assessments of cost-effectiveness and clinical utility will support translation into routine practice.

## 5. Conclusions

In conclusion, this study adds to the growing evidence that blood tau proteins (p-tau181, p-tau217 and p-tau231) may have substantial potential as a diagnostic and monitoring biomarker for Alzheimer’s disease. Elevated blood tau, consistent with tau-PET findings, supports its use as a surrogate marker of brain tau pathology. Validation in large-scale, longitudinal cohorts is still required; however, blood tau proteins could complement existing modalities, expand access to early detection, and enable more effective disease monitoring. Ultimately, integrating blood-based tau biomarkers into clinical practice may refine diagnostic frameworks and potentially improve the outcomes for individuals at risk of or living with AD.

## Figures and Tables

**Table 1:** General characteristics of studies.

Study (Author, Year)	Country	Analyzed Group	Number	Female *n* (%)	Age (M ± SD)	Cognitive Tests	Outcome Measures
Benedet et al., 2021 [16]	Canada	TRIAD: CU−	114	74 (64)	69.9 (9.4)	MMSE	29 (1.0)
		CU+	42	29 (69)	74.1 (7.7)		29 (1.0)
		MCI+	39	21 (53.8)	71.2 (7.7)		28 (2.0)
		AD	45	21 (46.7)	66.1 (9.7)		19 (6.0)
Doré et al., 2022 [7]	Australia, USA	CU	223	122 (54.7)	75.2 (5.70)	CDR-SB MMSE	0.0 (0.00) 29 (2.00)
		MCI	91	40 (44)	73.6 (7.99)		1.0 (1.50) 27 (4.00)
		Dementia	83	34 (41)	70.7 (7.91)		4.0 (1.00) 23 (4.00)
Ferrari-Souza et al., 2023 [17]	Canada	TRIAD: CU	62	43 (69.4)	70.7 (7.0)	MMSE	29.2 (1.2)
		MCI	25	15 (60)	71.3 (4.8)		28.0 (1.6)
		AD	7	3 (42.9)	71.2 (5.2)		23.4 (5.0)
Ferreira et al., 2023 [18]	Canada	CU−	108	67 (62)	68.2 (10.1)	MMSE MoCA	29.1 (1.11) 27.9 (1.82)
		CU+	30	20 (66.7)	72.5 (10.2)		29.0 (1.22) 28.3 (1.32)
		CI−	18	10 (43.5)	69.0 (11.8)		27.0 (2.63) 22.8 (5.36)
		CI+	69	43 (58.1)	68.4 (8.50)		24.6 (5.30) 20.8 (6.47)
Janelidze et al., 2020 [19]	Sweden	Cohort1: CU−	26	10	74 (71~78)	MMSE	29 (28~30)
		CU+	38	23	75 (71~79)		29 (29~30)
		MCI+	28	9	72 (69~78)		26 (24~29)
		AD+	38	17	73 (67~78)		21 (18~24)
Krishnadas et al., 2022 [20]	Australia	CU	1	0 (0)	76	MMSE CDR	29 0
		MCI	2	1 (50)	67/68		24/24 0.5/0.5
		AD	1	0 (0)	72		22 1
Leuzy et al., 2022 [21]	Sweden	CU−	137	64 (47)	72.57 (7.33)	MMSE	28.98 (1.21)
		CU+	49	26 (53)	72.83 (7.52)		28.67 (1.30)
		MCI+	58	32 (55)	71.79 (7.97)		26.69 (1.91)
		AD	63	35 (56)	73.06 (6.90)		19.74 (4.23)
Mendes et al., 2024 [8]	Switzerland	CU	33	21 (64)	68.9 (7.6)	MMSE	28.2 (1.3)
		MCI	67	33 (49)	72.9 (6.4)		26.1 (3.1)
		Dementia	14	7 (50)	71.1 (8.8)		20.1 (5.6)
Mielke et al., 2022 [9]	USA	CU	629	532 (45.8)	70.9 [48.4, 79.8]		
		MCI	88	65 (42.5)	80.8 [75.8, 86.1]		
		Dementia	13	2 (13.3)	83.5 [81.5, 85.0]		
Mielke et al., 2018 [5]	USA	CU	172	119 (69.2)	71.9 (9.5)		
		MCI	57	45 (79.0)	71.4 (10.7)		
		AD	40	23 (57.5)	67.7 (9.2)		
Moscoso et al., 2021 [11]	USA, Canada	ADNI: CN	359	191 (53.2)	74.7 (6.7)	MMSE	29 [24–30]
		MCI	518	227 (43.8)	72.8 (7.9)		28 [24–30]
		AD	186	78 (41.9)	75.1 (7.8)		23 [9–26]
Ni et al., 2023 [2]	China	CANDI: CN	21	14 (66.7)	56.1 (8.7)	MMSE CDR	28 [26, 29] 0
		MCI	61	37 (60.6)	62.5 (9.7)		21 [19, 26] 0.5
		AD	90	65 (72.2)	63.3 (8.8)		11 [7, 17] 1–2
Thijssen et al., 2021 [22]	USA	UCSF: NC	118	63 (53.4)	60.9 (18)	MMSE CDR	29 (1) 0 (0)
		MCI	99	44 (44.4)	65.5 (13)		27 (2) 2 (1)
		AD	58	33 (56.9)	65.3 (10)		19 (7) 6 (3)
Thijssen et al., 2020 [23]	USA et al.	HC	69	32 (46.4)	60.6 (22)	MMSE CDR-SB	29.0 (1) 0 (0)
		MCI	47	21 (44.7)	60.8(14)		26.8 (3) 2.0 (1)
		AD	56	33 (58.9)	65.0 (9)		20.3 (6) 4.8 (3)
Tissot et al., 2022 [14]	Canada	TRIAD: CU	162	102 (63)	69.4 (10.3)	MMSE CDR	No cognitive impairment
		MCI	60	27 (45)	70.3 (9.1)		≥26 0.5
		AD	32	16 (50)	64.9 (10.4)		<26 >0.5
Xiong et al., 2024 [12]	USA, Canada	ADNI: CN	863	382 (44.3)	72.7 (6.35)	ADAS-11 ADAS-13	5.33 [3.67, 7.33] 29.0 [29.0, 30.0]
		MCI	1068	626 (58.6)	72.8 (7.64)		9.67 [7.00, 13.0] 28.0 [26.0, 29.0]
		AD	409	230 (56.2)	74.9 (7.91)		19.0 [14.7, 23.0] 23.0 [21.0, 25.0]

Note: Data are expressed as the mean ± standard deviation (SD) or the median [interquartile range]. Abbreviations: AD: Alzheimer’s disease; ADAS: Alzheimer’s Disease Assessment Scale; ADNI: Alzheimer’s Disease Neuroimaging Initiative (ADNI) database; CANDI: China Aging and Neurodegenerative Disorder Initiative (CANDI) cohort; CDR-SB: Clinical Dementia Rating-Sum of Boxes; CN: cognitively normal; CU: cognitively unimpaired; CU−, Aβ-negative cognitively unimpaired; CU+, Aβ-positive cognitively unimpaired; MCI: mild cognitive impairment; MCI+, Aβ-positive mild cognitive impairment; MMSE, Mini-Mental State Examination; UCSF: University of California San Francisco (UCSF) cohort; TRIAD: Translational Biomarkers in Aging and Dementia (TRIAD) cohort.

**Table 2:** Plasma and PET tau biomarker results.

Study	Group	Blood Tau				Tau PET		
		Total Tau	p-Tau181	p-Tau217	p-Tau231	PET Tracer	SUVR Value or Correlation Measures	
Benedet et al., 2021 [16]	CU−		9.9 (4.4) *n* = 114			^18^F-MK6240	Spearman correlation coefficients (ρ) = 0.59 *n* = 293	
	CU+		14.8 (11.0) *n* = 42					
	MCI+		18.1 (8.1) * *n* = 39					
	AD		24.1 (9.6) *^,+^ *n* = 45					
Doré et al., 2022 [7]	CU			87.8 (67.8) *n* = 223		^18^F-MK6240	pTau217: correlated with meta Temporal ROI SUVR (Spearman ρ = 0.63, *p* < 10^−46^) 1.02 (0.23) *n* = 31	
	MCI			183.7 (141.0) * *n* = 91			1.48 (0.61) * *n* = 48	
	Dementia			229.9 (150.7) *^,+^ *n* = 83			1.93 (0.77) *^,+^ *n* = 64	
Ferrari-Souza et al., 2023 [17]	CU			0.05 (0.03) *n* = 62		^18^F-MK6240	pTau217: correlated with Temporal meta-ROI SUVR (*p* < 0.0001) 0.77 (0.10) *n* = 62	
	MCI			0.11 (0.06) * *n* = 25			1.04 (0.41) * *n* = 25	
	AD			0.40 (0.32) *^,+^ *n* = 7			1.81 (0.70) *^,+^ *n* = 7	
Ferreira et al., 2023 [18]	CU−		9.83 (4.35) *n* = 108	0.0487 (0.02)	12.7 (5.62)	^18^F-MK-6240	BraakI-II SUVR 0.948 (0.218) *n* = 108	pTau231: correlated with entorhinal SUVR (R^2^ = 0.17; *p* < 0.001 *) pTau217: correlated with entorhinal SUVR (R^2^ = 0.12; *p* < 0.001 *) pTau181: correlated with entorhinal SUVR (R^2^ = 0.16; *p* = 0.007)
	CU+		13.2 (5.49) *n* = 30	0.0905 (0.04)	21.1 (7.85)		1.28 (0.409) *n* = 30	
	CI−		12.9 (7.06) *n* = 18	0.09 (0.06)	14.1 (7.82)		1.10 (0.51) *n* = 18	pTau231: correlated with entorhinal SUVR (R^2^ = 0.18; *p* < 0.001 *) pTau217: correlated with entorhinal SUVR (R^2^ = 0.19; *p* < 0.001 *) pTau181: correlated with entorhinal SUVR (R^2^ = 0.17; *p* < 0.001 *)
	CI+		19.9 (8.42) *n* = 69	0.186 (0.12)	23.3 (10.1)		2.32 (0.88) *n* = 69	
Janelidze et al., 2020 [19]	Cohort1: CU−		1.3 [0.9–2.4]			^18^F-flortaucipir	*n* = 26 Temporal meta ROI SUVR Braak I–IV ROI, *p* = 3.3 × 10^−21^, 1.17 [1.10–1.20], Braak I–II ROI, *p* = 5.7 × 10^−19^, 1.06 [1.01–1.12] Braak III–IV ROI, *p* = 5.5 × 10^−21^, 1.17 [1.10–1.20] Braak V–VI ROI, *p* = 9.0 × 10^−17^, 1.05 [1.00–1.07] Inferior temporal cortex, *p* = 1.2 × 10^−19^, 1.21 [1.14–1.24]	
	CU+		1.9 [1.4–2.8] *				*n* = 38 1.15 [1.12–1.22] 1.09 [1.02–1.22] 1.16 [1.12–1.23] 1.04 [1.00–1.07] 1.21 [1.15–1.26]	
	MCI+		3.8 [2.5–5.7] *^,+^				*n* = 28 1.56 [1.25–1.80] 1.43 [1.25–1.78] 1.56 [1.24–1.82] 1.22 [1.06–1.28] 1.65 [1.30–1.90]	
	AD+		4.4 [3.3–6.4] *^,+^				*n* = 38 1.92 [1.60–2.29] 1.67 [1.50–1.75] 1.95 [1.60–2.31] 1.45 [1.18–1.64] 2.09 [1.70–2.54]	
Krishnadas et al., 2020 [20]	CU			High		^18^F-MK6240	*n* = 1 Mesial temporal SUVR: 1.14 Temporo-parietal SUVR: 1.37 * Rest of brain SUVR: 0.96	
	MCI			−(no test)/High			*n* = 2 2.73 */3.27 * 3.59 */4.01 * 1.89 */2.01 *	
	AD			High			*n* = 1 1.27 3.04 * 1.68 *	
Leuzy et al., 2022 [21]	CU−			1.29 (2.67)		^18^F-RO948	*n* = 137 EBM stage I: 0.97 (0.13) * EBM stage II: 1.27 (0.11) EBM stage III: 1.26 (0.12) EBM stage IV: 1.13 (0.11) EBM stage V: 1.18 (0.11)	
	CU+			3.22 (2.39)			*n* = 49 1.17 (0.21) * 1.42 (0.39) * 1.35 (0.36) * 1.16 (0.17) * 1.19 (0.12) *	
	MCI+			3.81 (2.14)			*n* = 58 1.38 (0.32) * 1.61 (0.45) * 1.55 (0.54) * 1.20 (0.18) * 1.20 (0.14) *	
	AD			8.01 (4.38)			*n* = 63 1.71 (0.36) * 2.62 (0.94) * 2.37 (0.91) * 1.69 (0.86) * 1.52 (0.43) *	
Mendes et al., 2024 [8]	CU		16.3 (7.6) *n* = 26	0.25 (0.3) *n* = 33	10.7 (4.2) *n* = 26	^18^F-flortaucipir	Total global SUVR 1.16 (0.17), *n* = 322	
	MCI		22.3 (10.6) *n* = 59	0.5 (0.42) * *n* = 67	13.3 (6.1) n = 60		1.35 (0.27), *n* = 66 *	
	Dementia		25.5 (11.1) * *n* = 10	0.7 (0.41) *^,+^ *n* = 14	12.2 (5.2) n = 10		1.57 (0.39), *n* = 12 *	
Mielke et al., 2022 [9]	CU		1.00 [0.80, 1.32]	0.14 [0.11, 0.19]		^18^F-flortaucipir	Temporal meta ROI (SUVR ≥ 1.29) 11.9% (55/462) Entorhinal cortex (SUVR ≥ 1.27) 8.7% (40/462)	
	MCI		1.61 [1.05, 2.47] *	0.24 [0.14, 0.39] *			28.1% (9/32) 25.0% (8/32)	
	Dementia		2.01 [1.45, 3.40] *	0.40 [0.15, 0.72] *			100% (1/1) 100% (1/1)	
Mielke et al., 2018 [5]	CU	5.9 (1.9) n = 172	6.4 (6.4) *n* = 172			^18^F-flortaucipir	Entorhinal cortex 1.1 (0.1) *n* = 172	
	MCI	5.9 (2.8) * n = 57	9.0 (13.9) *n* = 57				1.2 (0.2) *n* = 57 *	
	AD	7.2 (2.8) * n = 40	11.6 (4.1) * *n* = 40				1.8 (0.3) *n* = 40 *	
Moscoso et al., 2021 [11]	CN		13.6 [0.8–72.3]			^18^F-flortaucipir	**CN vs. MCI:** higher SUVR in Braak III–IV and V–VI regions in MCI	
	MCI		15.8 [1.6–69.6]				**MCI vs. AD:** higher SUVR in Braak III–IV and V–VI regions in AD	
	AD		23.2 [6.3–63.3]				**CN vs. AD:** higher SUVR in all regions in AD	
Ni et al., 2023 [2]	CN	2.840 [1.925, 3.839]	2.430 [1.565, 3.817]			^18^F-flortaucipir	p-tau181: correlated with global SUVR (r = 0.257, *p* < 0.0001)	
	MCI	2.693 [2.108, 3.743]	3.298 [1.863, 5.317]				p-tau181/t-tau ratio: correlated with global SUVR (r = 0.263, *p* < 0.0001)	
	AD	2.946 [2.390, 3.844]	5.883 [4.175, 7.451] *^,+^					
Thijssen et al., 2021 [22]	NC		0.9 (1)	0.17 (0.1)		^18^F-flortaucipir	Temporal meta ROI SUVR 1.1 (0.1) *n* = 8	
	MCI		1.2 (1) *	0.29 (0.3) *^,+^			1.4 (0.3) *n* = 46 *	
	AD		2.3 (1) *^,+^	0.72 (0.4) *^,+^			2.0 (0.4) *n* = 50 *^,+^	
Thijssen et al., 2020 [23]	HC		2.4 (3) *n* = 69			^18^F-flortaucipir	Cortical SUVR	
	MCI		3.7 (6) *n* = 47				1.2 (0) *n* = 31	
	AD		8.4 (4) *^,+^ *n* = 56				1.8 (0) *n* = 48 *^,+^	
Tissot et al., 2022 [14]	TRIAD: CU		11.3 (6.9) *n* = 162		15.4 (8.6) *n* = 162	^18^F-MK6240	Temporal meta ROI SUVR 1.1 (0.2) *n* = 162	
	MCI		16.1(8.6) * *n* = 60		18.1 (9.5) * *n* = 60		1.6 (0.8) * *n* = 60	
	AD		26.8(12.9) *^,+^ *n* = 32		27.6 (11.0) *^,+^ *n* = 32		2.6 (0.9) *^,+^ *n* = 32	
Xiong et al., 2024 [12]	ADNI: CN	2.52 [1.77, 3.11]	14.0 [9.85, 19.2]			^18^F-flortaucipir	1.18 [1.13, 1.23] *n* = 863	
	MCI	2.62 [1.76, 3.45]	17.1 [11.2, 24.4] *^,+^				1.22 [1.15, 1.37] * *n* = 1068	
	AD	2.82 [2.09, 3.84] *^,+^	23.0 [17.5, 27.8] *^,+^				1.53 [1.27, 1.80] *^,+^ *n* = 409	

Note: Data are expressed as the mean ± standard deviation (SD) or the median [interquartile range]; * *p*-value < 0.05 compared to CU or HC, ^+^ *p*-value < 0.05 compared to MCI. Abbreviations: AD, Alzheimer’s disease; CN, cognitively normal; EBM, event-based modeling; EBM stage I (entorhinal cortex, hippocampus, and amygdala), EBM II (temporal cortical regions), EBM IV (certain frontal regions); MCI, mild cognitive impairment; ROI, region of interest; p-tau181, tau phosphorylated at threonine 181; t-tau, total tau; SUVR: standard uptake value ratio.

## Data Availability

The data supporting the findings of this study are available from the corresponding author upon reasonable request.

## Supplementary Materials

The following supporting information can be downloaded at: https://www.mdpi.com/article/10.3390/ijms262110330/s1.

## Abbreviations

The following abbreviations are used in this manuscript:

AD	Alzheimer’s disease
ADAS	Alzheimer’s Disease Assessment Scale
ADNI	Alzheimer’s Disease Neuroimaging Initiative (ADNI) database
CANDI	China Aging and Neurodegenerative Disorder Initiative (CANDI) cohort
CDR-SB	Clinical Dementia Rating-Sum of Boxes
CN	cognitively normal
CSF	cerebrospinal fluid
CU	cognitively unimpaired
CU−	Aβ-negative cognitively unimpaired
CU+	Aβ-positive cognitively unimpaired
EBM	event-based modeling
MCI	mild cognitive impairment
MCI+	Aβ-positive mild cognitive impairment
MCSA	mayo clinic study of aging
MMSE	Mini-Mental State Examination
PET	positron emission tomography
p-tau181	tau phosphorylated at threonine 181
RoBANS	Risk of Bias Assessment Tool for Non-randomized Studies
ROI	region of interest
SUVR	standard uptake value ratio
TRIAD	Translational Biomarkers in Aging and Dementia (TRIAD) cohort
t-tau	total tau
UCSF	University of California San Francisco (UCSF) cohort
