# Supplementary material for: Blood-Based Tau as a Biomarker for Early Detection and Monitoring of Alzheimer’s Disease: A Systematic Review and Meta-Analysis
Source: Int J Mol Sci. 2025 Oct 23;26(21):10330. doi: 10.3390/ijms262110330 (PMC12607318; doi:10.3390/ijms262110330)
Supplement: Supplementary file 1 [file ijms-26-10330-s001.zip › ijms-3903052-supplementary.pdf]

Supplementary Figure S1. Leave-one-out sensitivity analyses

A. Plasma ptau181

Study excluded	Case, n	Control, n	SMD	95% CI	P value	I2
Benedet 2021 [1]	265	655	1.43	[1.27, 1.60]	<0.00001	73%
Ferreira 2023 [2]	241	661	1.52	[1.35, 1.69]	<0.00001	83%
Mendes 2024 [3]	300	743	1.56	[1.40, 1.71]	<0.00001	83%
Mielke 2018 [4]	270	597	1.69	[1.52, 1.86]	<0.00001	62%
Thijssen 2020 [5]	254	700	1.51	[1.35, 1.67]	<0.00001	83%
Thijssen 2021 [6]	252	651	1.57	[1.40, 1.74]	<0.00001	83%
Tissot 2022 [7]	278	607	1.48	[1.32, 1.65]	<0.00001	82%
Total	310	769	1.54	[1.38, 1.69]	<0.00001	80%

B. Plasma ptau217

Study excluded	Case, n	Control, n	SMD	95% CI	P value	I2
Dore 2022 [8]	211	458	2.01	[1.81, 2.21]	<0.00001	74%
Ferrari-souza 2023 [9]	287	619	1.78	[1.61, 1.94]	<0.00001	72%
Ferreira 2023 [2]	225	573	1.83	[1.65, 2.01]	<0.00001	84%
Leuzy 2022 [10]	231	544	1.77	[1.59, 1.95]	<0.00001	83%
Mendes 2024 [3]	280	648	1.85	[1.68, 2.02]	<0.00001	83%
Thijssen 2021 [6]	236	563	1.73	[1.56, 1.91]	<0.00001	80%
Total	310	769	1.54	[1.38, 1.69]	<0.00001	80%

C. Plasma ptau231

Study excluded	Case, n	Control, n	SMD	95% CI	P value	I2
Ferreira 2023 [2]	42	188	1.11	[0.76, 1.46]	<0.00001	82%
Mendes 2024 [3]	101	270	1.36	[1.11, 1.62]	<0.00001	0%
Tissot 2022 [7]	79	134	1.19	[1.89, 1.50]	<0.00001	85%
Total	111	296	1.25	[1.01, 1.49]	<0.00001	71%

D. PET – FTP

Study excluded	Case, n	Control, n	SMD	95% CI	P value	I2
Mendes 2024 [3]	90	180	3.84	[3.38, 4.30]	<0.00001	94%
Mielke 2018 [4]	62	330	2.29	[1.79, 2.78]	<0.00001	0%
Thijssen 2021 [6]	52	494	3.44	[3.04, 3.85]	<0.00001	96%
Total	102	502	3.25	[2.88, 3.62]	<0.00001	94%

E. PET – 18F-Mk6240

Study excluded	Case, n	Control, n	SMD	95% CI	P value	I2
Dore 2022 [8]	108	332	2.97	[2.66, 3.27]	<0.00001	91%
Ferrari-souza 2023 [9]	165	301	2.40	[2.14, 2.66]	<0.00001	95%
Ferreira 2023 [2]	103	255	2.61	[2.27, 2.94]	<0.00001	96%
Tissot 2022 [7]	140	201	2.15	[1.86, 2.45]	<0.00001	93%
Total	172	363	2.51	[2.25, 2.77]	<0.00001	94%

F. PET – all

Study excluded	Case, n	Control, n	SMD	95% CI	P value	I2
Dore 2022 [8]	273	971	0.70	[0.64, 0.75]	<0.00001	95%
Ferrari-souza 2023 [9]	330	948	0.71	[0.65, 0.76]	<0.00001	95%
Ferreira 2023 [2]	268	894	0.67	[0.61, 0.72]	<0.00001	93%
Leuzy 2022 [10]	274	865	0.84	[0.78, 0.90]	<0.00001	91%
Mendes 2024 [3]	325	680	0.73	[0.68, 0.79]	<0.00001	95%
Mielke 2018 [4]	297	830	0.72	[0.65, 0.79]	<0.00001	95%
Thijssen 2021 [6]	287	994	0.67	[0.61, 0.73]	<0.00001	95%
Tissot 2022 [7]	305	840	0.69	[0.63, 0.74]	<0.00001	94%
Total	337	1002	0.71	[0.66, 0.77]	<0.00001	95%

Supplementary Figure S2. Funnel plots assessing publication bias

A. Plasma tau in AD vs. controls

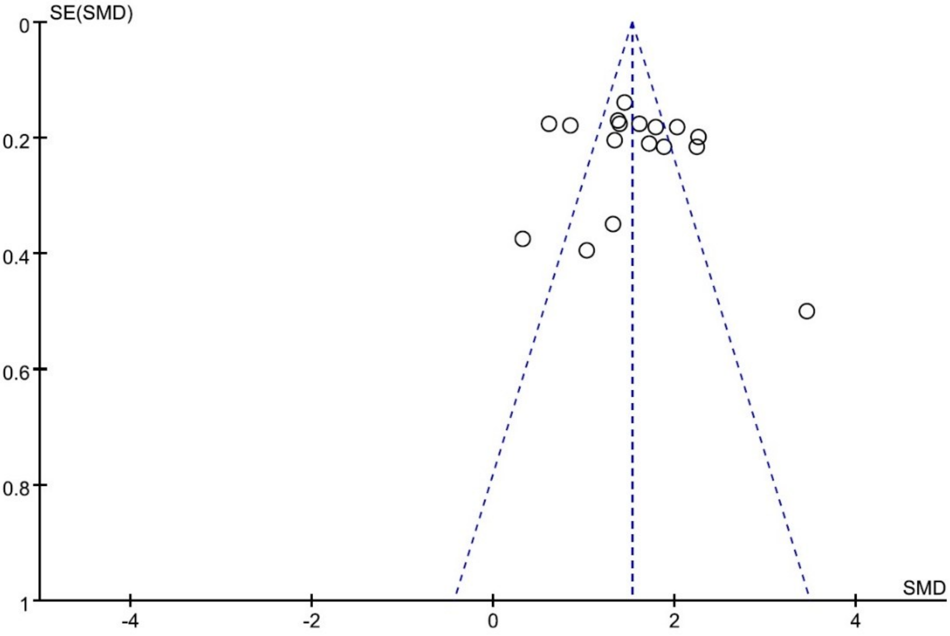

B. Plasma tau in MCI vs. controls

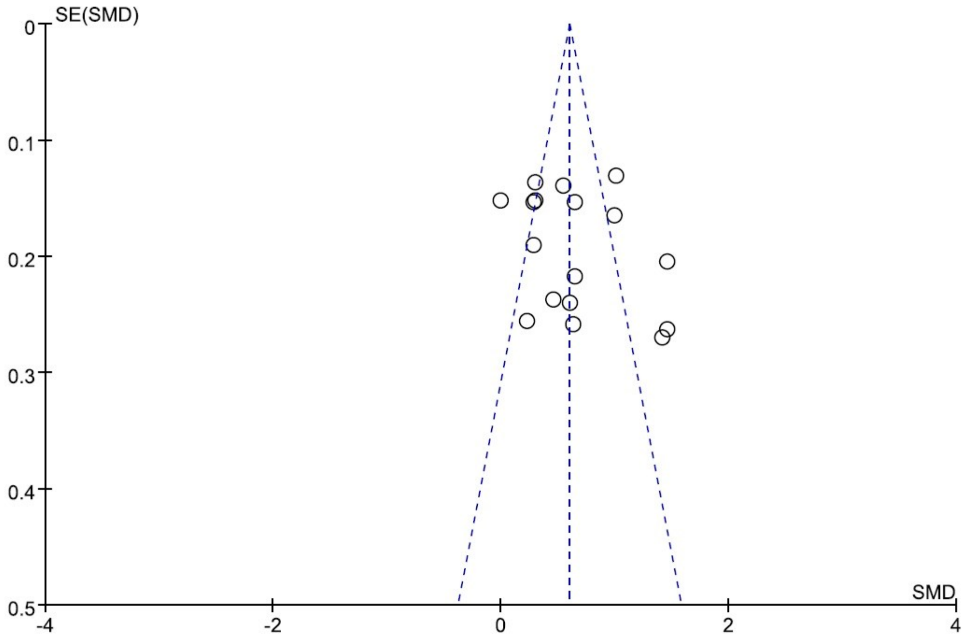

C. Plasma tau in AD vs. MCI

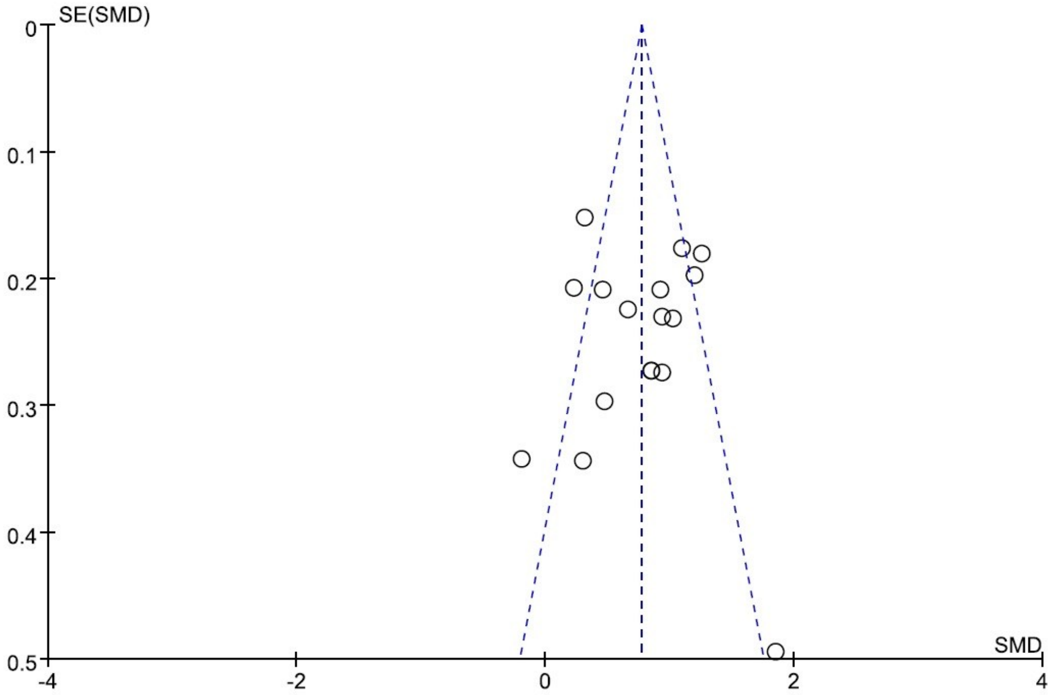

D. PET in AD vs. controls

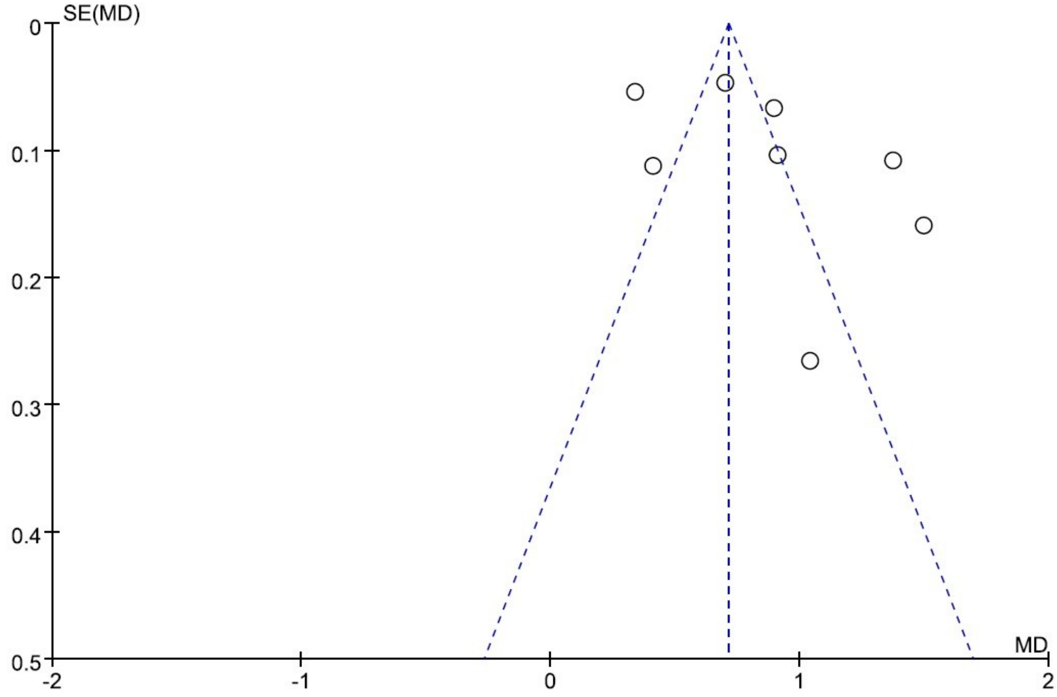

E. PET in MCI vs. controls

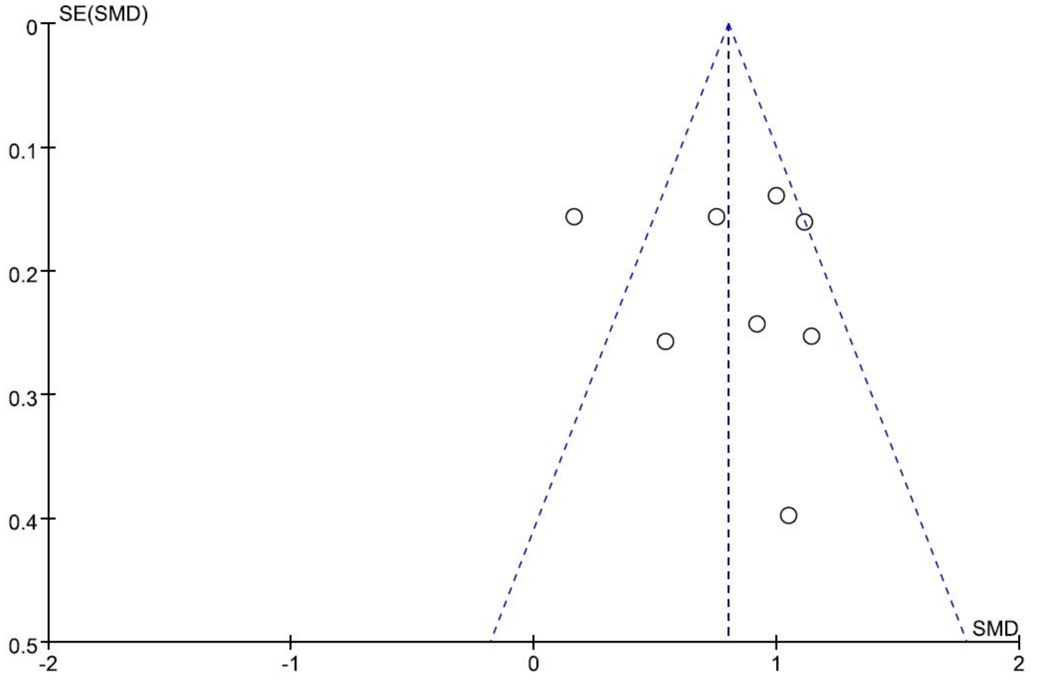

F. PET in AD vs. MCI

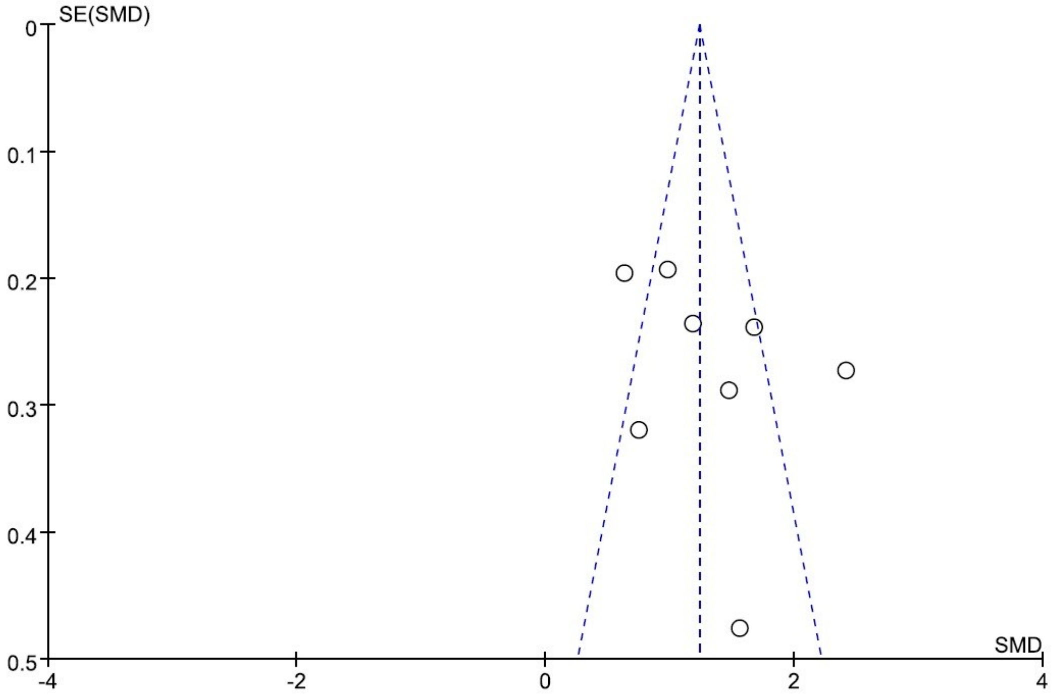

Supplementary Figure S3. Forest plots of meta-analysis of plasma tau biomarkers (overall)

A. AD vs controls

Study or Subgroup	AD Mean	AD SD	AD Total	Control Mean	Control SD	Control Total	Weight	Std. Mean Difference IV, Fixed, 95% CI
Benedet 2021 [16]	24.1	9.6	45	9.9	4.4	114	5.2%	2.24 [1.82, 2.67]
Dore 2022 [7]	229.9	150.7	83	87.8	67.8	223	12.2%	1.46 [1.18, 1.73]
Ferrari-Souza 2023 [17]	0.4	0.32	7	0.05	0.03	62	1.0%	3.46 [2.48, 4.44]
Ferreira 2023_ptau181	19.9	8.42	69	9.83	4.35	108	7.8%	1.60 [1.26, 1.95]
Ferreira 2023_ptau217	0.186	0.12	69	0.0487	0.02	108	7.4%	1.79 [1.43, 2.14]
Ferreira 2023_ptau231	23.3	10.1	69	12.7	5.62	108	8.4%	1.37 [1.04, 1.71]
Leuzy 2022 [21]	8.01	4.38	63	1.29	2.67	137	7.3%	2.03 [1.67, 2.39]
Mendes 2024_ptau181	25.5	11.1	10	16.3	7.6	26	1.6%	1.04 [0.27, 1.81]
Mendes 2024_ptau217	0.7	0.41	14	0.25	0.3	33	2.0%	1.32 [0.63, 2.00]
Mendes 2024_ptau231	12.2	5.2	10	10.7	4.2	26	1.7%	0.33 [-0.41, 1.06]
Mielke 2018_ptau181	11.6	4.1	40	6.4	6.4	172	7.5%	0.86 [0.50, 1.21]
Mielke 2018_total tau	7.2	2.8	40	5.9	1.9	172	7.7%	0.62 [0.27, 0.97]
Thijssen 2020 [23]	8.4	4	56	2.4	3	69	5.5%	1.71 [1.30, 2.13]
Thijssen 2021_ptau181 [22]	2.3	1	58	0.9	1	118	7.8%	1.39 [1.05, 1.74]
Thijssen 2021_ptau217 [22]	0.72	0.4	58	0.17	0.1	118	6.0%	2.25 [1.86, 2.65]
Tissot 2022_ptau181 [14]	26.8	12.9	32	11.3	6.9	162	5.2%	1.89 [1.47, 2.31]
Tissot 2022_ptau231 [14]	27.6	11	32	15.4	8.6	162	5.8%	1.35 [0.94, 1.75]
**Total (95% CI)**			**755**			**1918**	**100.0%**	**1.52 [1.43, 1.62]**

Heterogeneity: Chi² = 106.34, df = 16 (P < 0.00001); I² = 85%
Test for overall effect: Z = 30.80 (P < 0.00001)

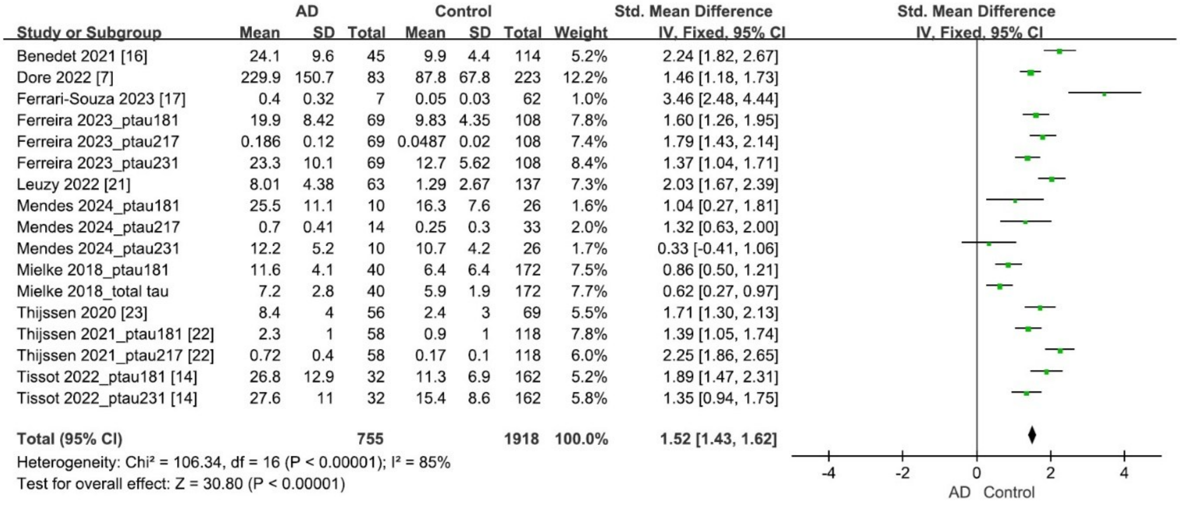

B. MCI vs controls

Study or Subgroup	MCI Mean	MCI SD	MCI Total	Control Mean	Control SD	Control Total	Weight	Std. Mean Difference IV, Fixed, 95% CI
Benedet 2021 [16]	18.1	8.1	39	9.9	4.4	114	4.5%	1.47 [1.07, 1.87]
Dore 2022 [7]	183.7	141	91	87.8	67.8	223	10.9%	1.01 [0.75, 1.26]
Ferrari-Souza 2023 [17]	0.11	0.06	25	0.05	0.03	62	2.7%	1.46 [0.94, 1.97]
Ferreira 2023_ptau181	12.9	7.06	18	9.83	4.35	108	2.8%	0.63 [0.13, 1.14]
Ferreira 2023_ptau217	0.09	0.06	18	0.0487	0.02	108	2.5%	1.42 [0.89, 1.95]
Ferreira 2023_ptau231	14.1	7.82	18	12.7	5.62	108	2.9%	0.23 [-0.27, 0.73]
Leuzy 2022 [21]	3.81	2.14	58	1.29	2.67	137	6.9%	0.99 [0.67, 1.32]
Mendes 2024_ptau181	22.3	10.6	59	16.3	7.6	26	3.2%	0.61 [0.14, 1.08]
Mendes 2024_ptau217	0.5	0.42	67	0.25	0.3	33	3.9%	0.64 [0.22, 1.07]
Mendes 2024_ptau231	13.3	6.1	60	10.7	4.2	26	3.3%	0.46 [-0.01, 0.93]
Mielke 2018_ptau181	9	13.9	57	6.4	6.4	172	7.9%	0.29 [-0.01, 0.59]
Mielke 2018_total tau	5.9	2.8	57	5.9	1.9	172	8.0%	0.00 [-0.30, 0.30]
Thijssen 2020 [23]	3.7	6	47	2.4	3	69	5.1%	0.29 [-0.08, 0.66]
Thijssen 2021_ptau181 [22]	1.2	1	99	0.9	1	118	9.9%	0.30 [0.03, 0.57]
Thijssen 2021_ptau217 [22]	0.29	0.3	99	0.17	0.1	118	9.6%	0.55 [0.28, 0.83]
Tissot 2022_ptau181 [14]	16.1	8.6	60	11.3	6.9	162	7.8%	0.65 [0.34, 0.95]
Tissot 2022_ptau231 [14]	18.1	9.5	60	15.4	8.6	162	8.1%	0.30 [0.01, 0.60]
**Total (95% CI)**			**932**			**1918**	**100.0%**	**0.60 [0.51, 0.68]**

Heterogeneity: Chi² = 86.70, df = 16 (P < 0.00001); I² = 82%
Test for overall effect: Z = 13.90 (P < 0.00001)

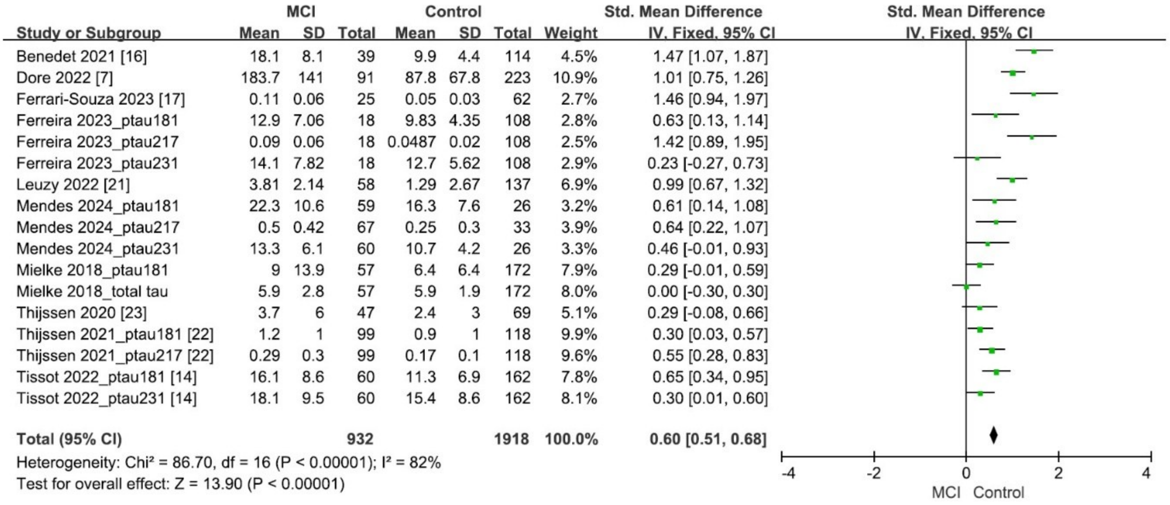

C. AD vs MCI

Study or Subgroup	AD Mean	AD SD	AD Total	MCI Mean	MCI SD	MCI Total	Weight	Std. Mean Difference IV, Fixed, 95% CI
Benedet 2021 [16]	24.1	9.6	45	18.1	8.1	39	6.0%	0.67 [0.22, 1.11]
Dore 2022 [7]	229.9	150.7	83	183.7	141	91	13.0%	0.32 [0.02, 0.62]
Ferrari-Souza 2023 [17]	0.4	0.32	7	0.11	0.06	25	1.2%	1.85 [0.88, 2.82]
Ferreira 2023_ptau181	19.9	8.42	69	12.9	7.06	18	4.1%	0.85 [0.32, 1.38]
Ferreira 2023_ptau217	0.186	0.12	69	0.09	0.06	18	4.1%	0.86 [0.33, 1.40]
Ferreira 2023_ptau231	23.3	10.1	69	14.1	7.82	18	4.0%	0.94 [0.40, 1.48]
Leuzy 2022 [21]	8.01	4.38	63	3.81	2.14	58	7.7%	1.20 [0.81, 1.58]
Mendes 2024_ptau181	25.5	11.1	10	22.3	10.6	59	2.6%	0.30 [-0.38, 0.97]
Mendes 2024_ptau217	0.7	0.41	14	0.5	0.42	67	3.4%	0.47 [-0.11, 1.05]
Mendes 2024_ptau231	12.2	5.2	10	13.3	6.1	60	2.6%	-0.18 [-0.85, 0.49]
Mielke 2018_ptau181	11.6	4.1	40	9	13.9	57	7.1%	0.23 [-0.17, 0.64]
Mielke 2018_total tau	7.2	2.8	40	5.9	2.8	57	6.9%	0.46 [0.05, 0.87]
Thijssen 2020 [23]	8.4	4	56	3.7	6	47	7.0%	0.93 [0.52, 1.34]
Thijssen 2021_ptau181 [22]	2.3	1	58	1.2	1	99	9.7%	1.09 [0.75, 1.44]
Thijssen 2021_ptau217 [22]	0.72	0.4	58	0.29	0.3	99	9.3%	1.26 [0.90, 1.61]
Tissot 2022_ptau181 [14]	26.8	12.9	32	16.1	8.6	60	5.6%	1.03 [0.58, 1.49]
Tissot 2022_ptau231 [14]	27.6	11	32	18.1	9.5	60	5.7%	0.94 [0.49, 1.39]
**Total (95% CI)**			**755**			**932**	**100.0%**	**0.77 [0.66, 0.88]**

Heterogeneity: Chi² = 51.66, df = 16 (P < 0.0001); I² = 69%
Test for overall effect: Z = 14.01 (P < 0.00001)

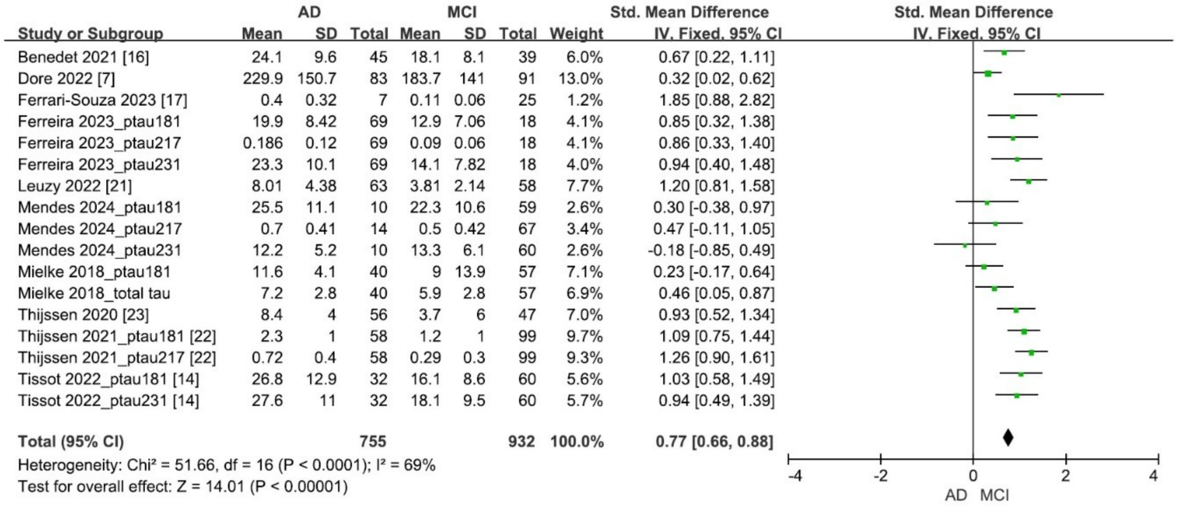

References

1. Benedet, A.L.; Milà-Alomà, M.; Vrillon, A.; Ashton, N.J.; Pascoal, T.A.; Lussier, F.; Karikari, T.K.; Hourregue, C.; Cognat, E.; Dumurgier, J.; et al. Differences between Plasma and Cerebrospinal Fluid Glial Fibrillary Acidic Protein Levels across the Alzheimer Disease Continuum. *JAMA Neurology* **2021**, *78*, 1471-1483, doi:10.1001/jamaneurol.2021.3671.
2. Ferreira, P.C.L.; Therriault, J.; Tissot, C.; Ferrari-Souza, J.P.; Benedet, A.L.; Povala, G.; Bellaver, B.; Leffa, D.T.; Brum, W.S.; Lussier, F.Z.; et al. Plasma p-tau231 and p-tau217 inform on tau tangles aggregation in cognitively impaired individuals. *Alzheimer's and Dementia* **2023**, *19*, 4463-4474, doi:10.1002/alz.13393.
3. Mendes, A.J.; Ribaldi, F.; Lathuiliere, A.; Ashton, N.J.; Janelidze, S.; Zetterberg, H.; Scheffler, M.; Assal, F.; Garibotto, V.; Blennow, K.; et al. Head-to-head study of diagnostic accuracy of plasma and cerebrospinal fluid p-tau217 versus p-tau181 and p-tau231 in a memory clinic cohort. *Journal of Neurology* **2024**, *271*, 2053-2066, doi:10.1007/s00415-023-12148-5.
4. Mielke, M.M.; Hagen, C.E.; Xu, J.; Chai, X.; Vemuri, P.; Lowe, V.J.; Airey, D.C.; Knopman, D.S.; Roberts, R.O.; Machulda, M.M.; et al. Plasma phospho-tau181 increases with Alzheimer's disease clinical severity and is associated with tau- and amyloid-positron emission tomography. *Alzheimers Dement* **2018**, *14*, 989-997, doi:10.1016/j.jalz.2018.02.013.
5. Thijssen, E.H.; La Joie, R.; Wolf, A.; Strom, A.; Wang, P.; Iaccarino, L.; Bourakova, V.; Cobigo, Y.; Heuer, H.; Spina, S.; et al. Diagnostic value of plasma phosphorylated tau181 in Alzheimer's disease and frontotemporal lobar degeneration. *Nature Medicine* **2020**, *26*, 387-+, doi:10.1038/s41591-020-0762-2.
6. Thijssen, E.H.; La Joie, R.; Strom, A.; Fonseca, C.; Iaccarino, L.; Wolf, A.; Spina, S.; Allen, I.E.; Cobigo, Y.; Heuer, H.; et al. Plasma phosphorylated tau 217 and phosphorylated tau 181 as biomarkers in Alzheimer's disease and frontotemporal lobar degeneration: a retrospective diagnostic performance study. *The Lancet Neurology* **2021**, *20*, 739-752, doi:10.1016/S1474-4422(21)00214-3.
7. Tissot, C.; Therriault, J.; Kunach, P.; A, L.B.; Pascoal, T.A.; Ashton, N.J.; Karikari, T.K.; Servaes, S.; Lussier, F.Z.; Chamoun, M.; et al. Comparing tau status determined via plasma pTau181, pTau231 and [(18)F]MK6240 tau-PET. *EBioMedicine* **2022**, *76*, 103837, doi:10.1016/j.ebiom.2022.103837.
8. Doré, V.; Doecke, J.D.; Saad, Z.S.; Triana-Baltzer, G.; Slemmon, R.; Krishnadas, N.; Bourgeat, P.; Huang, K.; Burnham, S.; Fowler, C.; et al. Plasma p217+tau versus NAV4694 amyloid and MK6240 tau PET across the Alzheimer's continuum. *Alzheimer's and Dementia: Diagnosis, Assessment and Disease Monitoring* **2022**, *14*, doi:10.1002/dad2.12307.
9. Ferrari-Souza, J.P.; Bellaver, B.; Ferreira, P.C.L.; Benedet, A.L.; Povala, G.; Lussier, F.Z.; Leffa, D.T.; Therriault, J.; Tissot, C.; Soares, C.; et al. APOEε4 potentiates amyloid β effects on longitudinal tau pathology. *Nature Aging* **2023**, *3*, 1210-1218, doi:10.1038/s43587-023-00490-2.
10. Leuzy, A.; Smith, R.; Cullen, N.C.; Strandberg, O.; Vogel, J.W.; Binette, A.P.; Borroni, E.; Janelidze, S.; Ohlsson, T.; Jögi, J.; et al. Biomarker-Based Prediction of Longitudinal Tau Positron Emission Tomography in Alzheimer Disease. *JAMA Neurology* **2022**, *79*, 149-158, doi:10.1001/jamaneurol.2021.4654.
